# Low concentrations of doxycycline attenuates FasL-induced apoptosis in HeLa cells

**DOI:** 10.1186/s40659-015-0025-8

**Published:** 2015-07-24

**Authors:** Jung Mi Yoon, Sushruta Koppula, Se Jong Huh, Sun Jin Hur, Chan Gil Kim

**Affiliations:** 1 Department of Biotechnology, Konkuk University, Chungju, 380-701 Republic of Korea; 2 Department of Animal Science and Technology, Chung-Ang University, Anseong, Gyeonggi 456-756 South Korea

**Keywords:** Apoptosis, Caspase, Cisplatin, FasL, Hydrogen peroxide, Tetracycline

## Abstract

**Background:**

Doxycycline (DC) has been shown to possess non-antibiotic properties including Fas/Fas Ligand (FasL)-mediated apoptosis against several tumor types in the concentration range of 10–40 µg/mL. However, the effect of DC in apoptotic signaling at much low concentrations was not studied.

**Methods:**

The present study investigated the attenuation effect of low dose of DC on FasL-induced apoptosis in HeLa cell by the methods of MTT assay, fluorescence microscopy, DNA fragmentation, flow cytometry analysis, and western blotting.

**Results and conclusion:**

In the present findings we showed that low concentration of DC (<2.0 µg/mL) exhibited protective effects against FasL-induced apoptosis in HeLa cells. FasL treatment to HeLa cells resulted in a concentration-dependent induction of cell death, and treatment with low concentrations of DC (0.1–2 µg/mL) significantly (p < 0.001) attenuated the FasL-induced cell death as measured by 3-(4,5-Dimethylthiazol-2-yl)-2,5-diphenyltetrazolium bromide (MTT) assay. Further, the FasL-induced apoptotic features in HeLa cells, such as morphological changes, DNA fragmentation and cell cycle arrest was also inhibited by DC (0.5 µg/mL). Tetracycline and minocycline also showed similar anti-apoptotic effects but were not significant when compared to DC, tested at same concentrations. Further, DC (0.01–16 µg/mL) did not influence the hydrogen peroxide- or cisplatin-induced intrinsic apoptotic pathway in HeLa cells. Protein analysis using Western blotting confirmed that FasL-induced cleavage/activation of caspase-8 and caspase-3, were inhibited by DC treatment at low concentration (0.5 µg/mL). Considering the overall data, we report for the first time that DC exhibited anti-apoptotic effects at low concentrations in HeLa cells by inhibition of caspase activation via FasL-induced extrinsic pathway.

**Electronic supplementary material:**

The online version of this article (doi:10.1186/s40659-015-0025-8) contains supplementary material, which is available to authorized users.

## Background

Apoptosis is a regulated physiological process essential for maintaining cellular homeostasis [1, 2]. The signaling events leading to apoptosis can be divided into two major pathways, the intrinsic mitochondrial and extrinsic death receptors [3, 4]. It was well known that caspase activation is a crucial step at which cells become committed to undergo apoptosis [5, 6]. In intrinsic pathway, a variety of extracellular and intracellular stress stimuli converge at the mitochondrial level, resulting in the translocation of cytochrome c (Cyt c) from the mitochondria to the cytoplasm. Cyt c binds the cytosolic adapter protein Apaf-1, which allows the recruitment and activation of an initiator caspase caspase-9. Active caspase-9 then cleaves and activates procaspase-3. The ‘apoptosome’, a complex formed by Cyt c, Apaf-1 and caspase-9, is a critical activator of effective caspase [7–12]. The mitochondrial apoptosis pathway is also partly regulated by the Bcl-2 family of proteins. Bcl-2 family members may be pro-apoptotic or anti-apoptotic [13].

Apoptosis mediated by extrinsic death receptors, such as Fas and FasL has been shown to play an important role in regulating apoptosis. Ligands bind to their cell surface receptors and through signaling transduction cascades, lead to the activation of an initiator caspase, caspase-8. Once caspase-8 is activated, it can process effector caspases such as caspase-3, -6, and -7, to induce a caspase signaling cascade [14–16]. Crosslinking of Fas with natural FasL initiates an apoptotic signal transduction cascade leading to cell death [17]. 

Tetracyclines including minocycline (MIN) and doxycycline (DC), in addition to their antimicrobial activities possess cytotoxic activity against several tumor cells [18, 19]. Particularly, DC, a semi-synthetic tetracycline characterized by the presence of a dimethylamino group at C4 and a hydroxy group at C5 is made by modifying the chemical structure of a naturally occurring tetracycline, so as to enhance its antibiotic activity. DC can inhibit MMP expression and cell proliferation in various types of cultured cell lines and in animal models [18, 20, 21]. DC-mediated anti-proliferative activity may be associated with the regulation of cell proliferation as well as its ability to inhibit MMP activity [20, 22]. Further, DC was also reported to induce apoptosis, decrease the invasion of tumor cells, and suppress the metastatic potential in breast cancer and melanoma cell lines at concentrations of 5–10 μg/mL [22, 23]. Thus, DC has been evaluated in preclinical cancer models and entered in early clinical trials in patients with malignant diseases [24].

Recently, research has focused on the possible non-anti-microbial effects of tetracycline and its derivatives, especially their anti-tumor functions such as inhibition of solid malignant tumor proliferation, invasion, metastasis and as well as the induction of apoptosis in cultured tumor cells [25–28]. The ability of tetracyclines including DC, to induce apoptosis was well reported in osteosarcoma, prostatic cancer cells and Jurkat T lymphocytes [18, 20, 29].

On the other hand, there is an increasing body of evidence suggesting that the tetracyclines possess anti-apoptotic properties [30]. It was found that MIN and DC increased the survival of hippocampal neurons following global brain ischemia in gerbils, and this protection was associated with reduced caspase-1 expression. In several models of neuronal injury, MIN was found to be protective against Huntington’s disease, traumatic brain injury and Parkinson’s disease by regulating caspase-1 and/or caspase-3 expression [31–33]. These findings suggested that the anti-apoptotic effects of tetracyclines were mediated via inhibition of caspase expression and by mitochondrial stabilization. Although the authors [30] expressed their view that DC might possibly exhibit similar effects, no report exists till date.

Earlier literature showed that DC possessed non-antibiotic properties including Fas/Fas Ligand (FasL)-mediated apoptosis against several tumor types in the concentration range of 10–40 µg/mL. However, the effect of DC in apoptotic signaling at much low concentrations was not studied. Here we report that the low concentrations of DC significantly attenuated FasL-induced apoptosis in HeLa cells by inhibition of caspase activation via FasL-induced extrinsic pathway.

## Results

### Effect of DC on FasL-induced apoptosis in HeLa and NIH3T3 cells

**Fig. 1:**
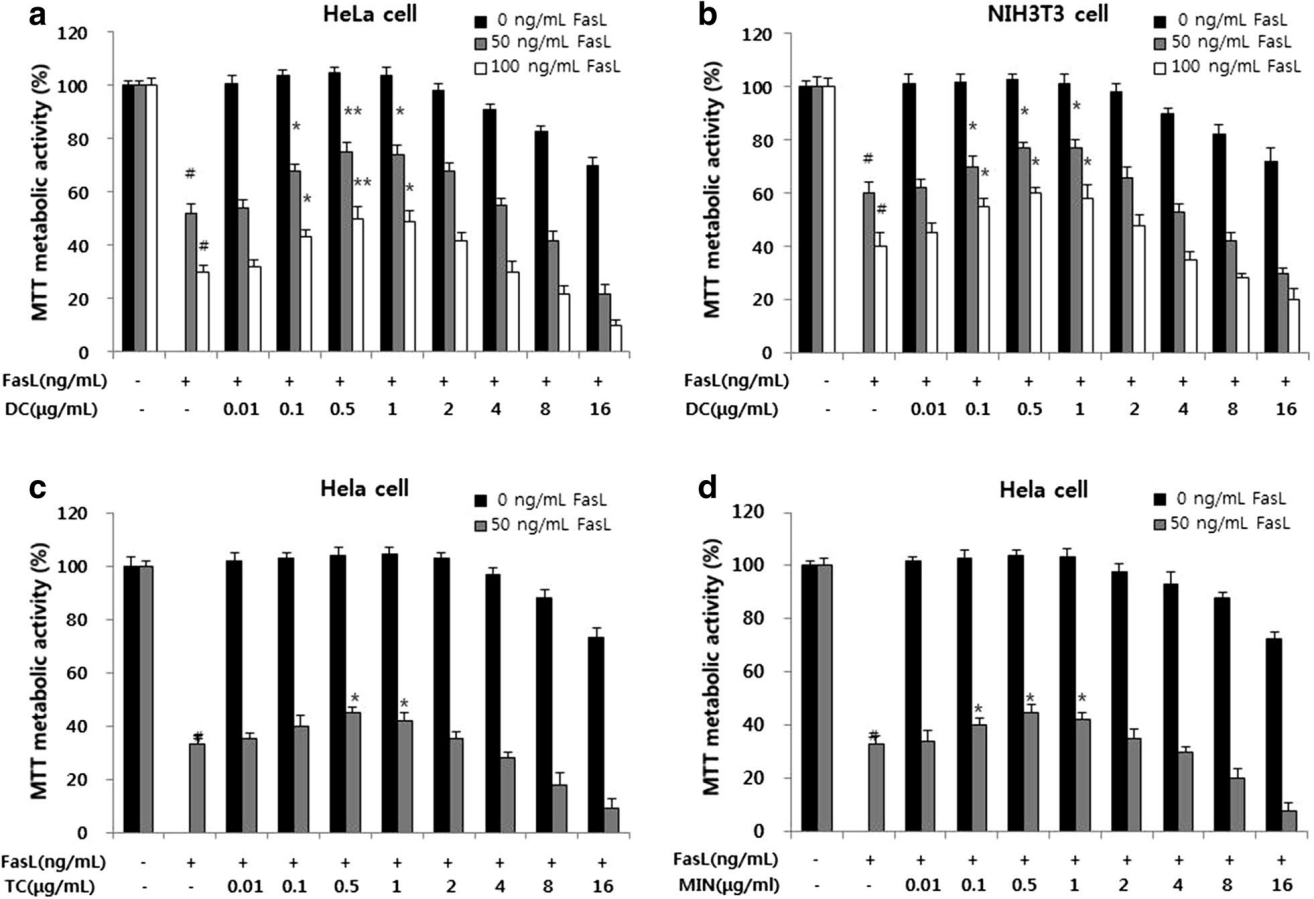
Effect of DC and other tetracyclines (TC and MIN) on FasL-induced apoptotic cell death. Cells were pretreated with indicated concentrations (0–16 µg/mL) of DC, TC and MIN for 12 h with or without FasL (50 or 100 ng/mL) for 24 h. The metabolic activity was measured by the MTT assay. Each point represents the mean ± SEM (n = 3). **a** Effect of DC on FasL-induced cell viability in HeLa cells. **b** Effect of DC on FasL-induced cell viability in NIH3T3 cells. **c** Effect of TC on FasL-induced cell viability in HeLa cells; **d** effect of MIN on FasL-induced cell viability in HeLa cells. The significance was determined by Student’s *t*-test (n = 3). ^#^ *p* < 0.05, compares with control group. *p < 0.05 and *p* < 0.01, compared with FasL treated groups. *DC* doxycycline, *TC* tetracycline, *MIN* minocycline.

The effects of DC at increasing concentrations (0.01–16 μg/mL) on FasL-induced apoptosis in HeLa and NIH3T3 cells measured by MTT assay were shown (Fig. 1). HeLa and NIH3T3 cells were pretreated with DC at indicated concentrations (0.01–16 μg/mL) for 12 h with or without FasL (50 or 100 ng/mL) treatment for 24 h. FasL induced significant reduction in cell viability at both 50 and 100 ng when compared to the control groups. Treatment with DC at low concentrations up to 0.5 µg/mL significantly and concentration dependently inhibited FasL-induced cell death in HeLa cells (Fig. 1a) and these effects were also confirmed by crystal violet assay (Additional file 1: Figure S1). However, DC at concentrations ranging from 4 to 16 µg/mL enhanced the FasL-induced cell death, reaching to a maximum at 16 µg/mL concentration. Similar pattern was observed with DC in FasL-induced cell death in NIH3T3 cells (Fig. 1b). Further to understand the effect of low dose of DC on FasL-induced cell death in other cancer cells we performed similar experiments using cancer cell lines such as MDA-MB-231 (human breast adenocarcinoma cells), LNCap (human prostate adenocarcinoma cells), U-87 MG (human glioblastoma cells), and TXM-1 (human melanoma cells). Although FasL-induced cell death was observed in other cancer cells lines, and DC attenuated this cell death the results were not significant at tested doses (Additional file 2: Figure S2). FasL-induced apoptotic cell death was significantly attenuated by low dose of DC (0.5 µg/mL) in only HeLa cancer cells when compared with the effects observed in other cancer cells.

### Effect of tetracycline and MIN on FasL-induced apoptosis in HeLa cells

To investigate the effect of tetracycline and MIN on FasL-induced apoptotic cell death, tetracycline and MIN at various concentrations (0.01–16 µg/mL) were incubated with FasL (50 ng/mL) in HeLa cells. Cell viability was measured by MTT assay. It was observed that both tetracycline (Fig. 1c) and MIN (Fig. 1d) showed similar pattern like DC. However, the concentration required to inhibit the FasL-induced cell death by tetracycline and MIN was much higher compared to the effect observed by DC (0.5 µg/mL). These results suggest that DC was efficient and significant (p < 0.01 at 0.5 µg/mL) in inhibiting the FasL-induced apoptotic cell death in HeLa cells when compared to tetracycline and MIN.

### Effect of DC on cisplatin- and oxidative stress (H_2_O_2_)-induced apoptosis

**Fig. 2:**
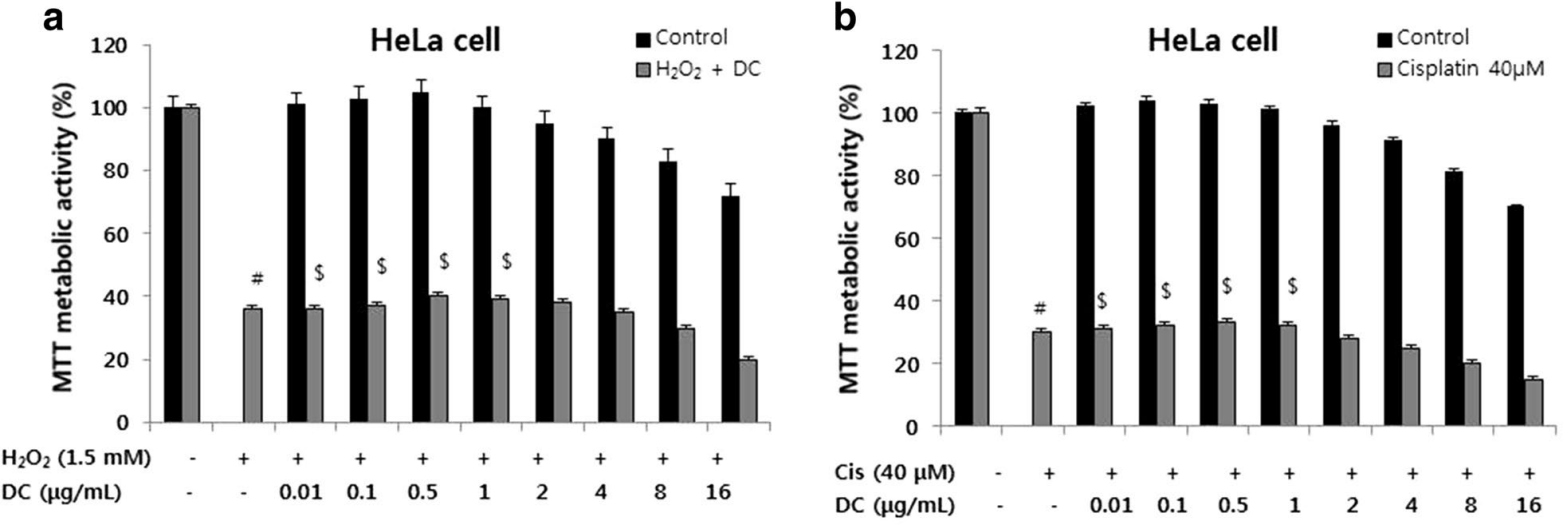
Effect of DC on hydrogen peroxide (H_2_O_2_)—or cisplatin-induced apoptotic cell death in HeLa cells. **a** HeLa cells were pretreated with indicated concentrations of DC (0.01–16 µg/mL) for 12 h with or without H_2_O_2_ (1.5 mM) for 24 h. **b** HeLa cells were pretreated with indicated concentrations of DC (0.01–16 µg/mL) for 12 h with or without cisplatin (40 µM) for 24 h. The cell viability was measured by the MTT assay. Each point represents the mean ± SEM (n = 3). The significance was determined by Student’s t-test. ^#^ *p* < 0.05 when compared with control group. ^$^No significant difference when compared with H_2_O_2_ or cisplatin treated groups. *DC* doxycycline.

Cisplatin and oxidative stress can cause cell death via intrinsic apoptotic pathway. Thus, to evaluate the effect of DC on intrinsic apoptosis, we used cisplatin- and H_2_O_2_-induced apoptosis models in HeLa cells. HeLa cells were incubated with various concentrations of DC with or without cisplatin or H_2_O_2_. Cell viability was measured by MTT assay. As shown in Fig. 2, H_2_O_2_ (1.5 mM) and cisplatin (40 µM) induced significant apoptotic cell death in HeLa cells. However, treatment with DC at various concentrations (0.01–16 µg/mL) in the presence of H_2_O_2_ (Fig. 2a) or cisplatin (Fig. 2b) did not show any improvement in cell viability in HeLa cells. These results indicated that DC at low concentrations did not influence the oxidative stress and cisplatin-mediated intrinsic apoptotic pathway, but inhibited the FasL-induced apoptotic cell death via extrinsic pathway.

### Effect of low concentrations of DC on FasL-induced morphological changes using DAPI staining

Initially, to select optimum concentrations of DC and FasL we performed the cell viability assay using MTT. We found that 0.5 µg/mL of DC did not exhibit any signs of toxicity but inhibited FasL-induced cytotoxicity significantly in HeLa cells. Also 50 ng/mL of FasL showed optimum (>45%) cytotoxicity (data not shown). Therefore for further apoptotic related experiments we used 0.5 µg/mL of DC and 50 ng/mL of FasL, respectively.

**Fig. 3:**
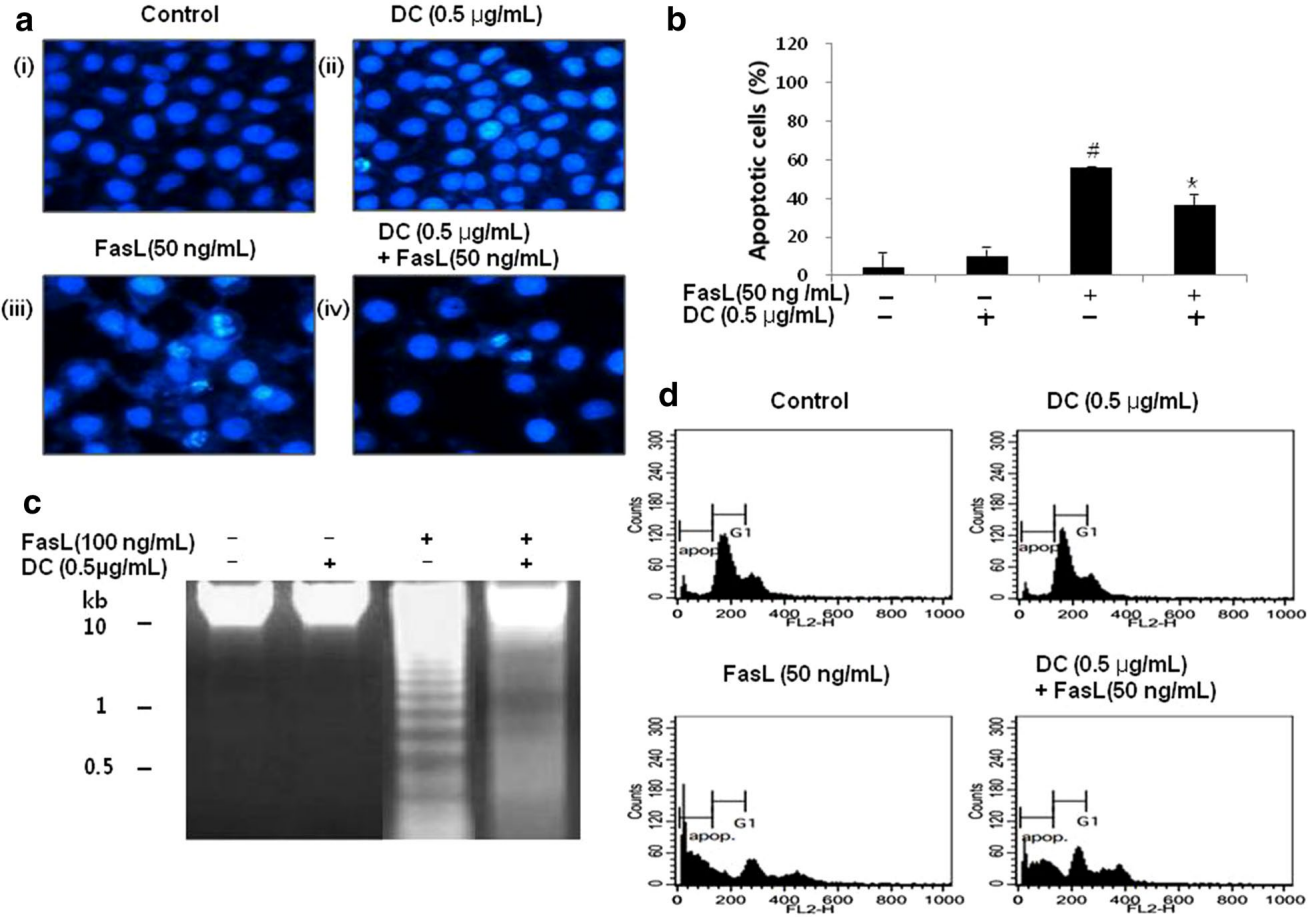
Effect of DC on morphological studies, DNA fragmentation and cell cycle arrest in FasL- induced apoptotic cell death in HeLa cells. HeLa cells were treated with indicated concentrations of DC (0.5 µg/mL) and FasL (50 ng/mL) as described in the methods. **a** The cells were fixed and stained with DAPI solution. The stained nuclei were the observed under a fluorescent microscope (×400). **b** The percentage of apoptotic cells was quantified. **c** DNA fragmentation assay. Same amount of genomic DNA (3 µg) extracted from cells was separated by 2.0% agarose gel electrophoresis, and visualized under UV light after staining with EtBr. *Marker* indicates a size marker of the DNA ladder. **d** To evaluate the degree of apoptosis reduced by DC, cells were evaluated by Flow Cytometry for sub-G1 DNA content (hypodilpoid DNA), which represents the cells undergoing apoptotic DNA degradation. Data are the mean ± SEM (n = 3). The significance was determined by Student’s t-test. ^#^ *p* < 0.05 compared with control group and **p < 0.01, compared with FasL treated group. *DC* doxycycline.

Further to understand the effect of DC on FasL-induced apoptosis morphologically in HeLa cells, we performed the DAPI staining. As shown in Fig. 3a, the nuclei of untreated control, DC treated alone and/or FasL-treated cells were stained with DAPI solution. Results revealed that control cells (Fig. 3a, i) and DC (0.5 µg/mL) treated cells (Fig. 3a, ii) displayed intact nuclear structure while cells treated with FasL (50 ng/mL) displayed apoptotic morphological characteristics, such as chromatin condensation and nuclear fragmentation in HeLa cells (Fig. 3a, iii). However, treatment with DC (0.5 µg/mL) to FasL treated cells restored the cell viability and morphological changes in HeLa cells (Fig. 3a, iv). Quantification data from counting over 200 cells (n = 3) revealed that FasL treated at 50 ng/mL induced cell death up to 50% (*p* < 0.05) and treatment with DC inhibited the cell death significantly (*p* < 0.05, Fig. 3b).

In addition, nucleosomal DNA ladder formation by 1.2% agarose gel electrophoresis was observed in HeLa cells treated with DC and/or FasL for 24 h. The results indicated that treatment with DC (0.5 µg/mL) alone did not affect the overall cell viability and FasL (100 ng/mL) alone treated cell showed DNA fragmentation. However, DC treatment to cells in the presence of FasL inhibited apoptosis (Fig. 3c). We further analyzed the degree of apoptotic cell death by Flow Cytometer. Cells treated with both DC and FasL resulted in inhibition of accumulation of HeLa cells at the sub-G1 phase (Fig. 3d). These data suggested that DC has ability to inhibit FasL-induced apoptosis. Further, percentages of cells in each phase were shown in Table 1.

**Table 1:** Percentage of cells in different cell cycle phases in FasL-induced HeLa cells treated with DC

	Control (%)	DC 0.5 µg/mL (%)	FasL 50 ng/mL (%)	DC 0.5 µg/mL + FasL 50 ng/mL (%)
Sub-G1	9.67 ± 0.89	9.29 ± 0.42	62.74 ± 3.84^#^	39.39 ± 1.58*
G1 phase	52.15 ± 2.54	56.59 ± 2.86	22.54 ± 1.64^#^	37.09 ± 2.18*
S phase	6.44 ± 1.03	7.86 ± 1.84	5.34 ± 0.68	12. 42 ± 1.04*
G2 phase	18.77 ± 1.04	17.93 ± 2.01	11.33 ± 1.26^#^	19. 74 ± 1.93*

Percentages of cells in each phase were shown. Data are represented as mean ± SEM (n = 3).

^#^p < 0.05 compared with DC treated cells and * p < 0.05 compared with FasL treated cells using students t test.

### Effect of low concentration of DC on caspase inhibition

**Fig. 4:**
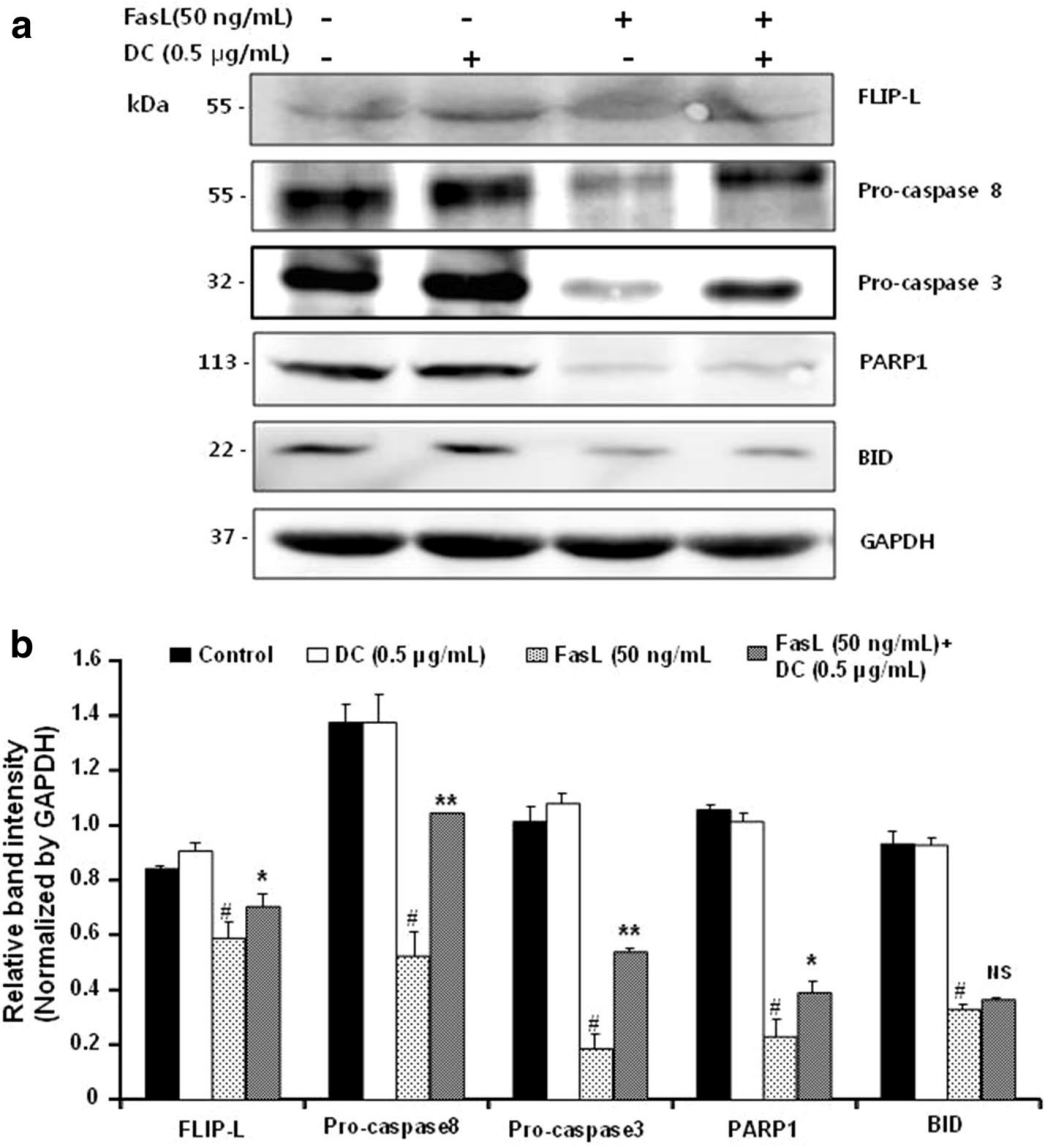
Inhibitory effect of low concentration of DC on the FasL-induced caspases cleavage in HeLa cells. HeLa cells were treated with the indicated concentrations of DC (0.5 µg/mL) and FasL (50 ng/mL) as described in the methods. **a** The cells were lysed and then cellular proteins were separated by sodium dodecyl sulfate (SDS)-polyacrylamide gels and transferred onto nitrocellulose membranes. The membranes were probed with the indicated antibodies. Proteins were visualized using an enhanced chemiluminescence (ECL) detection system. GAPDH was used as an internal control. **b** Quantification of protein expression levels of FLIP-L, pro-caspase 8, pro-caspase 3, PARP1 and BID normalized to GAPDH. Data are the mean ± SEM (n = 3). The significance was determined by Student’s t-test. ^#^ *p* < 0.05 compared with DC group. *p < 0.05 and **p < 0.01, compared with FasL treated group. *NS* not significant compared with DC treated group, *DC* doxycycline.

Caspases are well known to serve as an important mediator of apoptosis in extrinsic pathway. Therefore, to gain further insight into the mechanism by DC in inhibiting FasL-induced apoptosis, HeLa cells were pretreated with DC (0.5 µg/mL) before FasL (50 ng/mL) treatment for 24 h. Cell lysate was used to measure the expression of FLIP-L, pro-caspase-8, -3, PARP1, and BID. As shown in Fig. 4, treatment of cells with DC has no effect on the expression levels of Fas-signaling pathway related proteins except FLIP-L known to inhibit FasL-induced apoptosis. Treatment of cells with DC and FasL attenuated FasL-induced activation of caspase-8 and caspase-3. The relative band intensity of FLIP-L, pro-caspase 8, pro-caspase 3, PARP1 and BID, were measured by densitometric analysis and normalized with that of GAPDH (Fig. 4b). Taken together, as observed in Fig. 1a, low dose of DC treatment attenuated FasL-induced apoptosis in HeLa cells.

## Discussion

The present study reported for the first time that low concentrations of DC inhibited FasL-induced apoptosis in HeLa cells in several aspects. DC is a broad-spectrum antibiotic used clinically for over six decades. DC acts as such at the ribosomal level where it interferes with protein synthesis in prokaryotic cells. Recently, DC has been shown to possess anti-tumor functions, such as inhibition of tumor proliferation, invasion, metastasis as well as the induction of apoptosis [22, 23]. Reports from earlier studies related to the non-antibiotic properties of DC including apoptosis showed that DC in the concentration range of 10–40 µg/mL inhibited proliferation and induced apoptosis in several cancer cell lines [18, 20, 29, 34–36]. However, studies involving low concentrations of DC and its effect on apoptotic signaling were not elucidated. In the present investigation, we treated low concentrations of DC than commonly used and observed that DC at low concentrations inhibited FasL-induced apoptosis in HeLa cells.

FasL is a known inducer of apoptosis and is important in the regulation of several aspects of the immune system, including cytotoxic killing of cells potentially harmful to the organism such as virus-infected or tumor cells. DC at low concentrations (0.01–2 µg/mL) increased the cell viability significantly in FasL-induced cytotoxicity in cancer cell lines HeLa and NIH3T3 fibroblast cell lines. However at higher concentrations (4–16 µg/mL), DC enhanced the FasL-induced apoptotic cell death which was in agreement with the earlier studies [29, 34, 36]. Similar effects were observed with low concentrations of tetracycline and MIN, provided the anti-apoptotic effects were not significant as shown by DC in FasL-induced HeLa cells at similar concentrations. These results suggested that DC might be specific in significantly ameliorating Fas/FasL- mediated apoptosis. Although, the results depicted in this study showed the apoptotic level at one time point (12 h interval), examining the process of apoptosis at various time intervals might still deliver a clear picture in evaluating the changes during the process of apoptosis.

Apoptosis can be induced by diverse stimuli including reactive oxygen species (ROS) or cisplatin. H_2_O_2_ induces apoptosis by intrinsic pathway triggered by the release of Cyt c from mitochondria and results in the activation of the initiator caspase-9 which then cleaves and activates caspase-3 [37, 38]. Cisplatin, which crosslinks DNA in different pathways in turn activate apoptosis when repair proves impossible. In our present study, DC treated at indicated concentrations (0.01–16 µg/mL) did not influence the apoptotic cell death induced by H_2_O_2_ or cisplatin in HeLa cells. This further supports our notion that DC might act specifically via FasL-induced extrinsic apoptotic pathway.

Further, the morphological changes like chromatin condensation of nuclei and DNA fragmentation in FasL-induced in HeLa cells was inhibited markedly suggesting that low concentration of DC treatment (0.5 µg/mL) exhibited anti-apoptotic properties. Flow cytometric analysis in HeLa cells revealed that FasL induction for 24 h decreased the cells at the G1-S phase of the cell cycle. However treatment with low concentration of DC (0.5 µg/mL) attenuated the decreased cell percentage and reduced the portion of apoptotic cells.

It was well documented that oligomerization of Fas via binding of its cognate ligand FasL, induces a signaling cascade that culminates in the controlled degradation of cellular components [39]. The apical caspase-8 and caspase-9, together with the downstream effector caspase-3, have been documented to be crucial players in the mediation of death receptor-induced apoptosis [40]. In our study, the suppression of FasL-induced pro-caspase 8 and pro-caspase-3 protein expressions in HeLa cells was attenuated by treatment with low concentration of DC (0.5 µg/mL) suggesting that the effect of DC was mediated by the inhibition of caspase activation via FasL-induced extrinsic pathway. This might be attributed to a marked over-expression of FLIP-L observed by DC treatment.

## Conclusion

Our study was the first to demonstrate that low concentration of DC suppressed FasL-induced extrinsic apoptosis rather than cisplatin-induced intrinsic apoptosis by inhibition of caspase activation in HeLa cells.

## Methods

### Reagents

Dulbecco’s Modified Eagle’s Medium (DMEM), fetal bovine serum (FBS), penicillin and streptomycin were purchased from Hyclone (Logan, UT, USA). 3-(4, 5-dimethylthiazol-2-yl)-2, 5-diphenyltetrazolium bromide (MTT), 4′, 6-diamidino-2-phenylindole (DAPI), FasL, cisplatin, and all other chemicals were purchased from Sigma Aldrich Co. (MO, USA). The antibodies of PARP1 (cat # 9542), FLIP-L (cat # 8510), BID (cat # 8762), Caspase-3 (cat # 9668), -8 (cat # 9746), and GAPDH (cat # 2118) were purchased from Cell Signaling Technology Inc. (MA, USA).

### Cell culture

The cancer cell lines HeLa, MDA-MB-231 (human breast adenocarcinoma cells), LNCap (human prostate adenocarcinoma cells), U-87 MG (human glioblastoma cells), TXM-1 (human melanoma cells) and the fibroblast cell lines NIH3T3 were obtained from the American Type Culture Collection (ATCC, MD, USA) and maintained in DMEM supplemented with 10% heat-inactivated FBS and antibiotics (100 U/mL of penicillin, 100 μg/mL of streptomycin) in 5% CO_2_, 95% air and humidified atmosphere at 37℃.

### MTT and Crystal Violet assay

Cell viability was determined by MTT and Crystal Violet assay. HeLa cell and NIH3T3 cell were plated in triplicate at the concentration of 2.5 × 10^5^ cells/well on 96-well plate and pretreated with various concentrations of DC, tetracycline and MIN, respectively for 12 h and incubated with or without FasL for another 24 h. Similar conditions were followed for evaluating the effect of DC at various concentrations in hydrogen peroxide (H_2_O_2_; 1.5 mM)- and cisplatin (40 μM)-induced cytotoxicity in HeLa cells. For MTT assay, the medium was discarded and 100 μL of DMEM medium containing MTT (0.5 mg/mL) was added to each well, followed by incubation for 2 h at 37°C After incubation, the medium were discarded, 100 μL dimethyl sulfoxide (DMSO) was added to dissolve the MTT formazan. For Crystal Violet assay, the medium was discarded and stained with 30 μL of 0.05% Crystal Violet in 70% Methanol to each well, followed by incubation for 30 min at room temperature. The optical density for MTT and Crystal Violet assay were measured at 550 and 570 nm, respectively.

### DAPI staining

After treatment with DC or FasL, the cells were harvested, washed in ice-cold PBS and fixed with 4% paraformaldehyde in PBS for 10 min at room temperature. The fixed cells were washed with PBS and stained with a DAPI (300 nM) solution containing 0.2% TritonX-100 for 10 min at room temperature. The cells were washed twice with PBS and analyzed via a fluorescence microscope (Carl Zeiss, Oberkochen, Germany).

### DNA fragmentation assay

After treatment with DC or FasL, the cells were lysed in a buffer containing 10 mM Tris–HCl, pH 7.4, 150 mM NaCl, 5 mM EDTA, and 0.5% Triton X-100 for 20 s at room temperature. The lysates were vortexed and cleared by centrifugation at 3,000 rpm for 30 min at 4°C. The DNA in the supernatant was extracted using ethanol and analyzed electrophoretically on 2.0% agarose gels.

### Distribution of cells in the cell cycle phases

Pretreated cells with DC or FasL were collected, washed with cold PBS and fixed in 75% ethanol at 4°C overnight. Cells were stained with PI (2 µg/mL) in PBS containing 0.2% triton X-100 for 20 min at room temperature. Flow cytometric analyses were carried out using a flow cytometer.

### Western blotting

After treatment of DC or FasL, cells were harvested and washed twice in PBS at 4°C. Total cells were lysed in lysis buffer [40 mM Tris (pH8.0), 120 mM NaCl, 0.5% NP-40, 0.1 mM sodium orthovanadate, 2 μg/mL aprotinin, 2 μg/mL leupeptin, and 100 μg/mL phenymethylsulfonyl fluoride]. Equal amounts of protein extracts were subjected to 10–12% SDS–polyacrylamide gels, and transferred to Nitrocellulose membrane. Immunoblot analyses were performed using rabbit anti-mouse antibodies that recognize FLIP-L (1:1,000), Caspase 8 (1:500), Caspase 3 (1:500), PARP1 (1:1,000), BID (1:500), and GAPDH (1:1,000) in PBST buffer (80 mM Na2HPO4; 20 mM NaH2PO4; 100 mM NaCl; 0.1% Tween-20) + 1% BSA, followed by incubation with secondary antibody sheep anti-mouse or anti-rabbit HRP (1:2,500) in the same buffer. Detection was performed by enhanced chemiluminescence (ECL) kit according to the manufacturer’s instructions (Amersham Corp., Arlington Heights, IL, USA).

### Statistical analysis

Data are summarized as mean ± SEM (n = 3). The statistical analysis of the results was performed by the students *t* test using GraphPad Sigma-Plot Software (Systat Software Inc, CA, USA). In all experiments, *p* values less than 0.05 were considered statistically significant.

### Additional files

Additional file 1: **Figure S1.** Effect of DC on FasL-induced apoptotic cell death. HeLa cells were pretreated with indicated concentrations (0.01-16 µg/mL) of DC for 12h with or without FasL (150 ng/ml) for 24h. The cell viability was measured by the crystal violet assay. Each point represents the mean±S.E.M. (n=3). The significance was determined by Student’s *t*-test. ^#^ *p* < 0.05, compares with control groups. *p < 0.05, compared with FasL treated groups. DC: Doxycycline.

Additional file 2: **Figure S2.** Effect of DC on FasL-induced apoptotic cell death in many cancer cell lines. Cells were pretreated with indicated concentrations (0.5 µg/mL) of DC for 12h with or without FasL (100 ng/ml) for 24h. The cells viability were measured by the MTT assay. Tested cell lines were MDA-MB-231 (human breast adenocarcinoma cells), LNCap (human prostate adenocarcinoma cells), U-87 MG (human glioblastoma cells), and TXM-1 (human melanoma cells). Each point represents the mean ± S.E.M. (n=3).

## Abbreviations

DC: doxycycline
FasL: Fas Ligand
Cyt c: cytochrome c
MIN: minocycline
MTT: 3-(4,5-dimethylthiazol-2-yl)-2,5-diphenyltetrazolium bromide
DAPI: 6-diamidino-2-phenylindole

## References

[1] Reed JC (2000). Mechanisms of apoptosis. Am J Pathol.

[2] Sayers TJ (2011). Targeting the extrinsic apoptosis signaling pathway for cancer therapy. Cancer Immunol Immunother.

[3] MacKenzie SH, Clark AC (2008). Targeting cell death in tumors by activating caspases. Curr Cancer Drug Targets.

[4] Repický A, Jantová S, Milata V (2008). Signal pathways of cell proliferation and death as targets of potential chemotherapeutics. Ceska Slov Farm.

[5] Lynch DH, Ramsdell F, Alderson MR (1995). Fas and FasL in the homeostatic regulation of immune responses. Immunol Today.

[6] Thompson CB (1995). Apoptosis in the pathogenesis and treatment of disease. Science.

[7] Thorburn A (2004). Death receptor-induced cell killing. Cell Signal.

[8] Slee EA, Harte MT, Kluck RM, Wolf BB, Casiano CA, Newmeyer DD (1999). Ordering the cytochrome c-initiated caspase cascade: hierarchical activation of caspases-2, -3, -6, -7, -8, and -10 in a caspase-9-dependent manner. J Cell Biol.

[9] Lee H-J, Lee H-J, Lee E-O, Ko S-G, Bae H-S, Kim C-H (2008). Mitochondria-cytochrome C-caspase-9 cascade mediates isorhamnetin-induced apoptosis. Cancer Lett.

[10] Thornberry NA, Lazebnik Y (1998). Caspases: enemies within. Science.

[11] Oltvai ZN, Milliman CL, Korsmeyer SJ (1993). Bcl-2 heterodimerizes in vivo with a conserved homolog, Bax, that accelerates programmed cell death. Cell.

[12] Sedlak TW, Oltvai ZN, Yang E, Wang K, Boise LH, Thompson CB (1995). Multiple Bcl-2 family members demonstrate selective dimerizations with Bax. Proc Natl Acad Sci USA.

[13] Green DR, Kroemer G (2004). The pathophysiology of mitochondrial cell death. Science.

[14] Saxena N, Yadav P, Kumar O (2013). The Fas/Fas ligand apoptotic pathway is involved in abrin-induced apoptosis. Toxicol Sci.

[15] Neumann L, Pforr C, Beaudouin J, Pappa A, Fricker N, Krammer PH (2010). Dynamics within the CD95 death-inducing signaling complex decide life and death of cells. Mol Syst Biol.

[16] Peter ME, Budd RC, Desbarats J, Hedrick SM, Hueber A-O, Newell MK (2007). The CD95 receptor: apoptosis revisited. Cell.

[17] Ashkenazi A, Dixit VM (1998). Death receptors: signaling and modulation. Science.

[18] Fife RS, Rougraff BT, Proctor C, Sledge GW (1997). Inhibition of proliferation and induction of apoptosis by doxycycline in cultured human osteosarcoma cells. J Lab Clin Med.

[19] Gilbertson-Beadling S, Powers EA, Stamp-Cole M, Scott PS, Wallace TL, Copeland J (1995). The tetracycline analogs minocycline and doxycycline inhibit angiogenesis in vitro by a non-metalloproteinase-dependent mechanism. Cancer Chemother Pharmacol.

[20] Fife RS, Sledge GW, Roth BJ, Proctor C (1998). Effects of doxycycline on human prostate cancer cells in vitro. Cancer Lett.

[21] Uitto VJ, Firth JD, Nip L, Golub LM (1994). Doxycycline and chemically modified tetracyclines inhibit gelatinase A (MMP-2) gene expression in human skin keratinocytes. Ann N Y Acad Sci.

[22] Fife RS, Sledge GW (1995). Effects of doxycycline on in vitro growth, migration, and gelatinase activity of breast carcinoma cells. J Lab Clin Med.

[23] Duivenvoorden WC, Hirte HW, Singh G (1997). Use of tetracycline as an inhibitor of matrix metalloproteinase activity secreted by human bone-metastasizing cancer cells. Invasion Metastasis.

[24] Putnam JB, Light RW, Rodriguez RM, Ponn R, Olak J, Pollak JS (1999). A randomized comparison of indwelling pleural catheter and doxycycline pleurodesis in the management of malignant pleural effusions. Cancer.

[25] Sapadin AN, Fleischmajer R (2006). Tetracyclines: nonantibiotic properties and their clinical implications. J Am Acad Dermatol.

[26] Iwasaki H, Inoue H, Mitsuke Y, Badran A, Ikegaya S, Ueda T (2002). Doxycycline induces apoptosis by way of caspase-3 activation with inhibition of matrix metalloproteinase in human T-lymphoblastic leukemia CCRF-CEM cells. J Lab Clin Med.

[27] Hidalgo M, Eckhardt SG (2001). Development of matrix metalloproteinase inhibitors in cancer therapy. J Natl Cancer Inst.

[28] Lokeshwar BL, Selzer MG, Zhu B-Q, Block NL, Golub LM (2002). Inhibition of cell proliferation, invasion, tumor growth and metastasis by an oral non-antimicrobial tetracycline analog (COL-3) in a metastatic prostate cancer model. Int J Cancer.

[29] Liu J, Kuszynski CA, Baxter BT (1999). Doxycycline induces Fas/Fas ligand-mediated apoptosis in Jurkat T lymphocytes. Biochem Biophys Res Commun.

[30] Yrjanheikki J, Tikka T, Keinanen R, Goldsteins G, Chan PH, Koistinaho J (1999). A tetracycline derivative, minocycline, reduces inflammation and protects against focal cerebral ischemia with a wide therapeutic window. Proc Natl Acad Sci USA.

[31] Chen M, Ona VO, Li M, Ferrante RJ, Fink KB, Zhu S (2000). Minocycline inhibits caspase-1 and caspase-3 expression and delays mortality in a transgenic mouse model of Huntington disease. Nat Med.

[32] Sanchez Mejia RO, Ona VO, Li M, Friedlander RM (2001). Minocycline reduces traumatic brain injury-mediated caspase-1 activation, tissue damage, and neurological dysfunction. Neurosurgery.

[33] Du Y, Ma Z, Lin S, Dodel RC, Gao F, Bales KR (2001). Minocycline prevents nigrostriatal dopaminergic neurodegeneration in the MPTP model of Parkinson’s disease. Proc Natl Acad Sci USA.

[34] Son K, Fujioka S, Iida T, Furukawa K, Fujita T, Yamada H (2009). Doxycycline induces apoptosis in PANC-1 pancreatic cancer cells. Anticancer Res.

[35] Tolomeo M, Grimaudo S, Milano S, La Rosa M, Ferlazzo V, Di Bella G (2001). Effects of chemically modified tetracyclines (CMTs) in sensitive, multidrug resistant and apoptosis resistant leukaemia cell lines. Br J Pharmacol.

[36] Mouratidis PXE, Colston KW, Dalgleish AG (2007). Doxycycline induces caspase-dependent apoptosis in human pancreatic cancer cells. Int J Cancer.

[37] Nagata M, Yamamoto H, Shibasaki M, Sakamoto Y, Matsuo H (2000). Hydrogen peroxide augments eosinophil adhesion via beta2 integrin. Immunology.

[38] Enari M, Sakahira H, Yokoyama H, Okawa K, Iwamatsu A, Nagata S (1998). A caspase-activated DNase that degrades DNA during apoptosis, and its inhibitor ICAD. Nature.

[39] Suda T, Takahashi T, Golstein P, Nagata S (1993). Molecular cloning and expression of the Fas ligand, a novel member of the tumor necrosis factor family. Cell.

[40] Strasser A, O’Connor L, Dixit VM (2000). Apoptosis signaling. Annu Rev Biochem.

