# Supplementary figures and images for: Low concentrations of doxycycline attenuates FasL-induced apoptosis in HeLa cells
Source: Biol Res. 2015 Jul 24;48:38. doi: 10.1186/s40659-015-0025-8 (PMC4511997; doi:10.1186/s40659-015-0025-8)

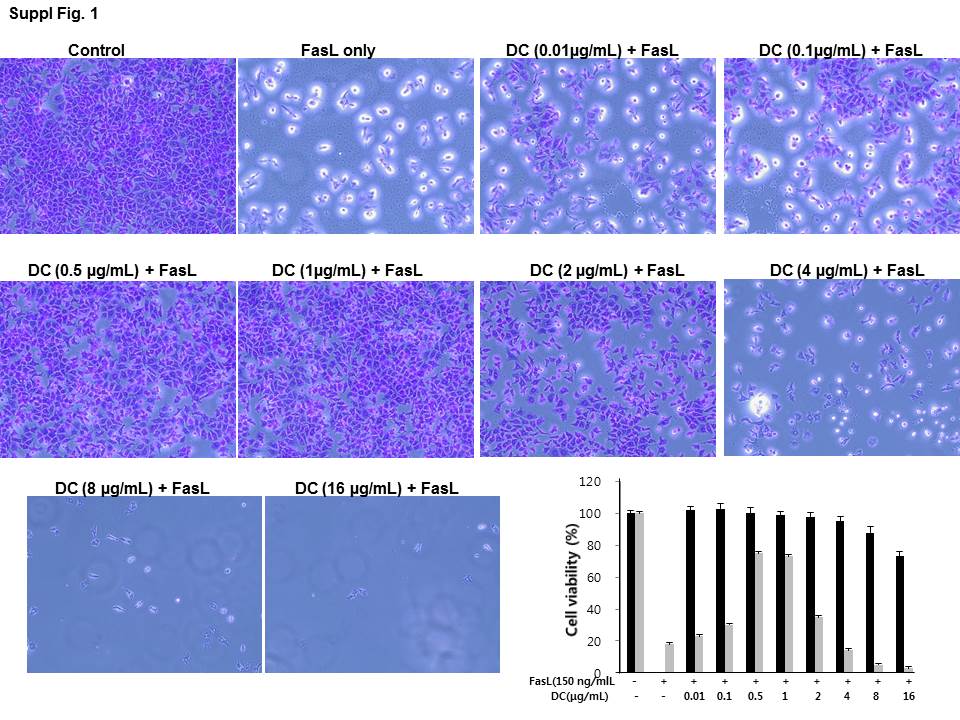

Supplement: Supplementary file 1 — Figure S1. Effect of DC on FasL-induced apoptotic cell death. HeLa cells were pretreated with indicated concentrations (0.01-16 µg/mL) of DC for 12h with or without FasL (150 ng/ml) for 24h. The cell viability was measured by the crystal violet assay. Each point represents the mean±S.E.M. (n=3). The significance was determined by Student’s t-test. # p < 0.05, compares with control groups. *p < 0.05, compared with FasL treated groups. DC: Doxycycline. [file 40659_2015_25_MOESM1_ESM.jpg]

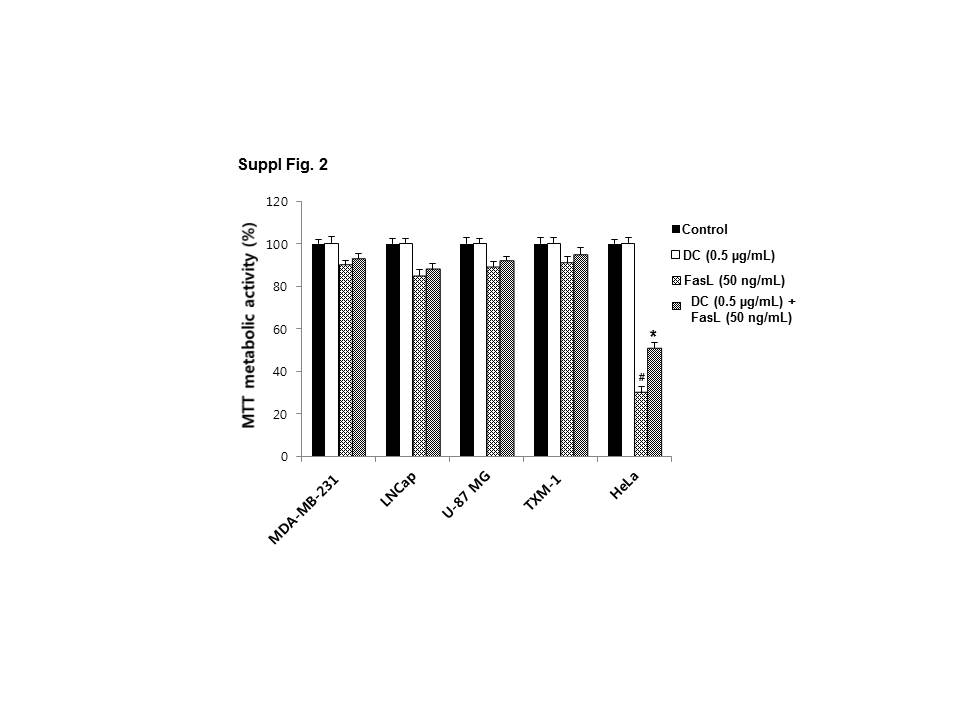

Supplement: Supplementary file 2 — Figure S2. Effect of DC on FasL-induced apoptotic cell death in many cancer cell lines. Cells were pretreated with indicated concentrations (0.5 µg/mL) of DC for 12h with or without FasL (100 ng/ml) for 24h. The cells viability were measured by the MTT assay. Tested cell lines were MDA-MB-231 (human breast adenocarcinoma cells), LNCap (human prostate adenocarcinoma cells), U-87 MG (human glioblastoma cells), and TXM-1 (human melanoma cells). Each point represents the mean ± S.E.M. (n=3). [file 40659_2015_25_MOESM2_ESM.jpg]
